# Bibliometric and visualized analysis of scientific publications on rehabilitation of rotator cuff injury based on web of science

**DOI:** 10.3389/fpubh.2023.1064576

**Published:** 2023-02-17

**Authors:** Yu Hu, Linfeng Wu, Lin He, Xiaozhou Luo, Linzhe Hu, Yuchan Wang, Xin Zhao

**Affiliations:** ^1^Department of Rehabilitation Medicine, The Third People's Hospital of Chengdu, Affiliated Hospital of Southwest Jiaotong University, Chengdu, Sichuan, China; ^2^Department of Orthopedics, The First People's Hospital of Longquanyi District, Chengdu, Sichuan, China; ^3^Center of Rehabilitation Medicine, West China Hospital, Sichuan University, Chengdu, Sichuan, China

**Keywords:** rehabilitation, physical therapy, rotator cuff injury, bibliometric analysis, visualized analysis

## Abstract

**Background:**

Since the discovery of rehabilitation as an intervention for rotator cuff injury, its impact on the recovery of rotator cuff injury has attracted crucial attention, and the number of related studies is increasing worldwide. There were no bibliometric and visualized analysis studies in this field. This study aimed to investigate the research hotpots and trends in the rehabilitation of rotator cuff injury *via* bibliometric and visualized analysis and to identify the future development of clinical practice.

**Method:**

The publications regarding rehabilitation of rotator cuff injury from inception to December 2021 were obtained from the Web of Science Core Collection database. The trends of publications, co-authorship and co-occurrence analysis and visualized analysis were carried out using Citespace, VOSviewer, Scimago Graphica software, and R Project.

**Results:**

A total of 795 publications were included in this study. The number of publications significantly increased yearly. The United States published the highest number of related papers and the papers published by the United States had the highest citations. The University of Laval, the University of Montreal and Keele University were the top 3 most contributive institutions. Additionally, the *Journal of Shoulder and Elbow Surgery* was the journal with the highest number of publications. The most common keywords were “rotator cuff”, “rehabilitation”, “physical therapy”, “management”, and “telerehabilitation”.

**Conclusion:**

The total number of publications has shown a steady upward trend. The cooperation between countries globally was still relatively lacking, and therefore it is necessary to strengthen cooperation between different countries and regions to provide conditions for multi-center, large sample, and high-quality research. In addition to the relatively mature rehabilitation of rotator cuff injury such as passive motion or exercise therapy, telerehabilitation has also attracted much attention with the progress of science.

## Introduction

Rotator cuff injury (RCI) is one of the leading causes of pain and dysfunction of the shoulder, resulting in limited activities of daily living and range of motion (ROM) of the shoulder (1). Orthopedic surgeons mentioned that rotator cuff lesions are the main cause of shoulder-related disability and that the numbers of surgery are on the rise (2). There was one study that noted a 141% increase in rotator cuff reconstruction in the United States (US) within 10 years (3) and between 30 and 50% of patients with RCI are over the age of 50 (4). There are approximately 4.5 million patients with shoulder pain every year in the US (4). Over two-thirds of them receive therapy due to rotator cuff tears at working age. In total 54% of asymptomatic patients aged over 60 have experienced either an incomplete or complete rotator cuff tear on magnetic resonance imaging (MRI) (5).

Injury and degeneration are two common mechanisms of RCI (6). The acute tear is usually caused directly by injury and this type of tear can occur in isolation or with other shoulder injuries (6). Additionally, the pathology of rotator cuff injuries is often found in tendon degeneration, impingement, and repetitive overhead activities (5). Most chronic shoulder pain is caused by repeated impingement of the rotator cuff at the acromion (5). The early manifestations are local edema and hemorrhage of the rotator cuff, and then develop Tendonitis with localized fibrosis (5). If the impact factor exists for a long time, it will eventually lead to rotator cuff tears (7). The rate of natural self-healing of RCI is inconstant due to the limited blood supply around the tendon (8). The size of the tear may develop over time and tendon retraction, and muscle atrophy will occur in uncured RCI, which may cause the shoulder function to become worse and eventually have negative effects on the quality of life and health status (9).

Currently, surgery is the common method for treating RCI. Narvy et al. have identified many common treatments such as surgery have a positive effect on RCI (2). However, one recent study indicated that the rate of retear after rotator cuff repair was roughly 20%, which resulted from varieties of factors such as insufficient repair or unsuitable rehabilitation plan after surgery (10). Rehabilitation is an integral factor in recovery from RCI. Studies have shown that both passive and active joint mobility training and strength training can effectively relieve stiffness and increase strength (11). Rehabilitation is critical to preserve the integrity of the rotator cuff and prevent stiffness (11). Lee et al. indicated that exercise from limited passive movement to continuous passive motion has a positive effect on enhancing strength and avoiding retear during the immobilization period of 4–6 weeks after rotator cuff repair (12). Traditional rehabilitation, especially post-operative rehabilitation programs, is often used to rebuild patients with shoulder dysfunction. However, the impact of different types of rehabilitation programs and the timing of rehabilitation interventions on RCI is controversial. For example, the study by Brislin et al. showed that early postoperative rehabilitation can reduce the adhesion of the shoulder joint and promote the recovery of shoulder joint function (13), but the study by Parsons et al. believes that early exercise may affect the rotator cuff heal (14). Parsons et al. believed that a relatively conservative 6-week suspension of the affected limb would not increase the occurrence of joint stiffness while promoting the healing of the rotator cuff (14). For these reasons, it is critical to determine the impact of rehabilitation on RCI.

First proposed in 1969, bibliometrics is the discipline of applying mathematical and statistical methods to quantitatively analyze information. It can be used to evaluate the contributions of countries, institutions, journals, and authors to specific research topics, and to identify research hotspots and trends in a certain field (15). Also, information gleaned from bibliometrics can provide evidence for the development of clinical guidelines (16). Although there have been systematic reviews and meta-analyses that provided a general overview of the application of rehabilitation on RCI, there are currently no bibliometric studies summarizing the large amount of previous literature to show the knowledge structure and development trends of rehabilitation on RCI. In addition, exploring the impact of rehabilitation on RCI has contributed to changes in the number of publications in this field, and it is vital for researchers or clinicians to understand this trend. However, there are currently no bibliometric studies related to the rehabilitation on RCI to provide the progress and overall trends in this field. Therefore, the purpose of this paper is to summarize the past three decades of research in the field of rehabilitation on RCI using the bibliometrics research method, to draw the visualized maps using CiteSpace, VOSviewer software, and R Project, and to quantitatively show the development, hotspots and development trends of this research field.

## Materials and methods

### Search strategy

**Figure 1:**
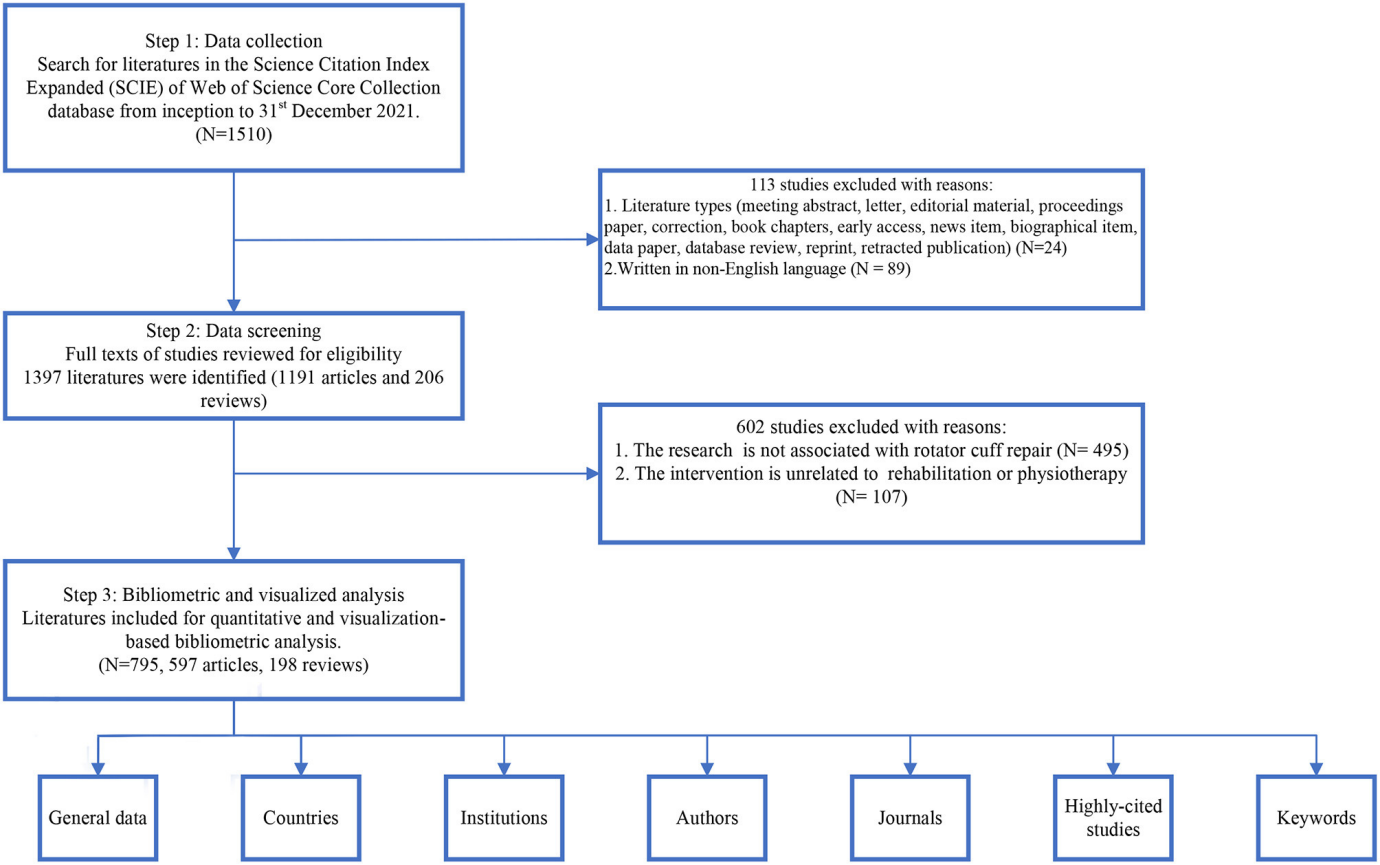
The flowchart of literature selection.

The data included in this study were obtained from literature in the Science Citation Index Expanded (SCIE) of the Web of Science (WOS) Core Collection (WOSCC) database from inception to 31 December 2021. An entire online search was accomplished on 14 July 2022. The keywords searched were established according to the medical subject heading (Mesh) terms in PubMed. The search strategy was as follows: TS = (Rehabilitation OR Physiotherapy OR “Physical therapy^*^” OR Physiotherapy (Techniques) OR Physiotherapies (Techniques) OR “Techniques, Physical Therapy” OR “Group Physiotherapy” OR Therapy, Physical) AND TS = (“Cuff Injury” OR “Rotator cuff” OR “Tear, Rotator Cuff” OR “Tears, Rotator Cuff”) AND TS = (Shoulder). Only peer-reviewed articles and reviews related to the rehabilitation of RCI that were written in English were included in this study. The flowchart of literature selection is shown in Figure 1.

### Data extraction and analysis

After searching in the SCIE of the WOSCC database, we obtained basic information including the number of publications, publication years, keywords, etc., and then imported these data into EndNote X9 for deduplication and literature screening. The included literature data were tabulated using Microsoft Excel 2016, and a linear regression analysis of the year and number of publications was constructed to evaluate the time trend graph of annual publications. We used VOSviewer (1.6.18) for co-occurrence network analysis with countries, institutions, authors and journals. The size of nodes in the VOSviewer map represents the number of publications, the connections between nodes represent the strength of relationships or collaborations, and the color of each circle represents the year of publication or cluster (17). In addition, Citespace (6.1.2) was used to show the co-occurrence network of keywords, the map of clusters, the timeline view of keywords clusters and the keywords with the strongest citation bursts. In the maps generated by the software, the connections between nodes represent the relevance of collaboration or co-citation. Different colors represent different citation years. The software can also identify research trends and research hotspots in specific fields by analyzing burst keywords (18). The Scimago Graphica (1.0.24) was used for geospatial visualization. The “bibliometrix” and “ggplot” packages in the R Project for Statistical Computing were used to create the heatmaps of country and keywords distribution over time.

## Results

### Number of global publications and growth trend

**Figure 2:**
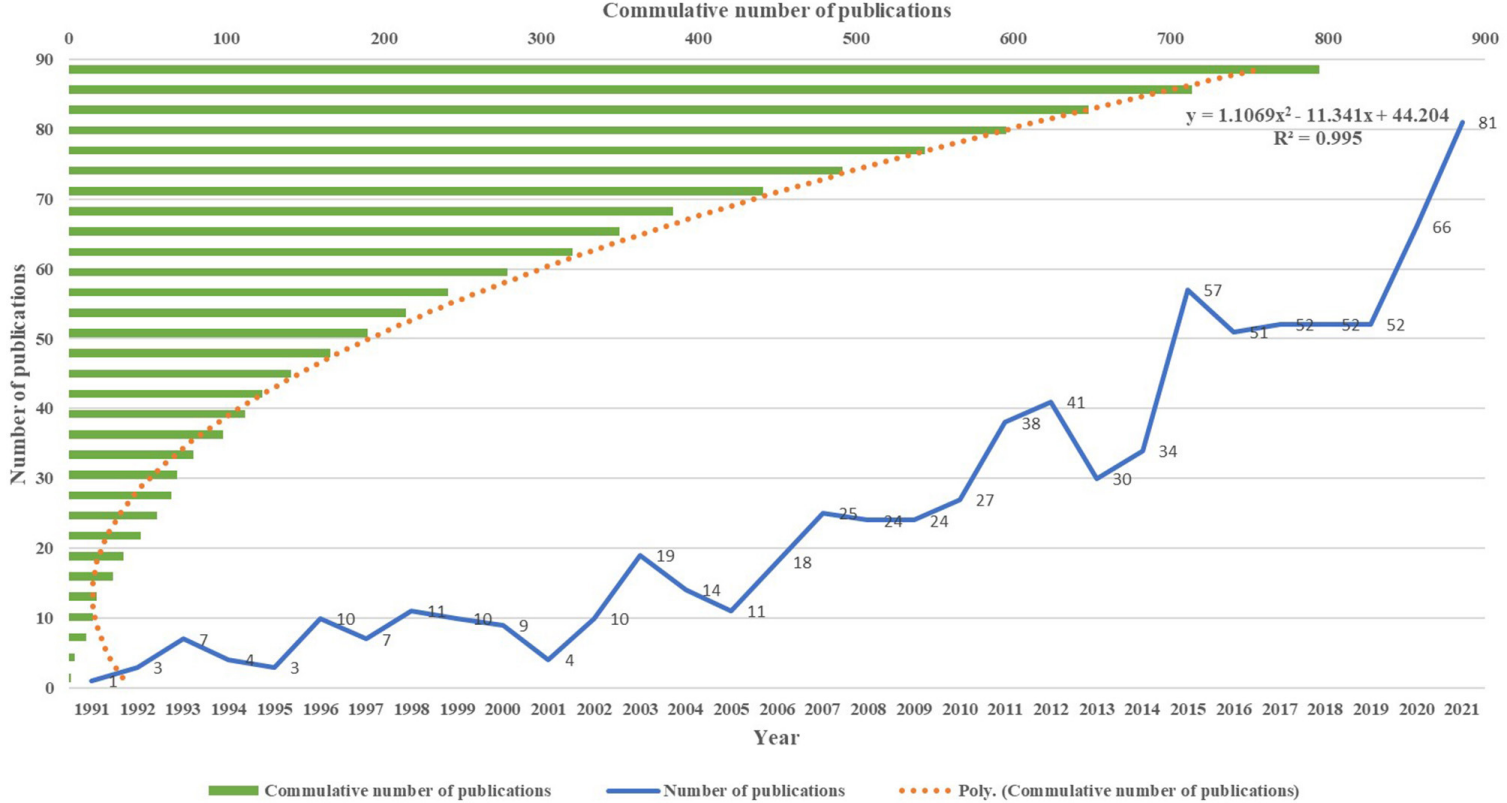
The trend of publication outputs from 1991 to 2021 on the rehabilitation of rotator cuff injury.

According to the search from the WOS database, from inception to December 2021, a total of 795 publications finally met the inclusion criteria, including 597 original articles, accounting for 75.1%. The result in Figure 2 shows that the earliest paper on the rehabilitation of RCI was published in 1991 by Chard, MD, which showed that around 70% of shoulder pain involves RCI and that less than 40% of patients receive rehabilitation (19). As shown in Figure 2, there is a significant upward trend in the number of publications worldwide. According to the development trend of this research field, the number of publications is divided into three stages. The first stage was from 1991 to 2001. During this stage, the number of published papers was relatively stable every year, basically less than 10 papers. The second stage was from 2002 to 2014. During this period, the number of published papers showed a steady and slow growth trend. The annual number of papers increased from 10 in 2002 to 34 in 2014. The third stage was from 2015 to 2022, and the annual number of publications shows a rapid growth trend. More than 50% of papers (*n* = 411) were published at this stage, with an average annual publication number of nearly 60, and the peak publication year was 2021 (*n* = 81). However, it is worth noting that from 2016 to 2019, the number of publications remained stable, with an average annual publication number of approximately 52. In the present study, linear regression analysis demonstrated a significant correlation (R2 = 0.995) between the annual number of publications and years over the recent three decades. Additionally, the average total citation per year was higher than most years in 2001, 2009, and 2013, which illustrated that the publications in these years not only hold high outputs but also have high value (Table 1).

**Table 1:** The number of publications and citations from 1991–2021.

**Year**	**N**	**Mean TC per publication**	**Mean TC per year**	**Year**	**N**	**Mean TC per publication**	**Mean TC per year**
1991	1	11	0.35	2007	25	40.6	2.71
1992	3	18.67	0.62	2008	24	43.21	3.09
1993	7	85.14	2.94	2009	24	74.88	5.76
1994	4	29.5	1.05	2010	27	42.63	3.55
1995	3	102.33	3.79	2011	38	45.03	4.09
1996	10	47.6	1.83	2012	41	44.44	4.44
1997	7	91.71	3.67	2013	30	54.3	6.03
1998	11	66.36	2.77	2014	34	36.79	4.60
1999	10	11.6	0.5	2015	57	24.74	3.53
2000	9	41	1.86	2016	51	26	4.33
2001	4	107	5.1	2017	52	15.44	3.09
2002	10	56.6	2.83	2018	52	13.13	3.28
2003	19	51.95	2.73	2019	52	8.18	2.73
2004	14	57.86	3.21	2020	66	7.77	3.88
2005	11	84	4.94	2021	81	2.27	2.27
2006	18	48.06	3				

TC, total citations; Mean TC per Publication, the average number of citations per publication; Mean TC per Year, the average number of citations per article per year.

### Contributions of countries

**Figure 3:**
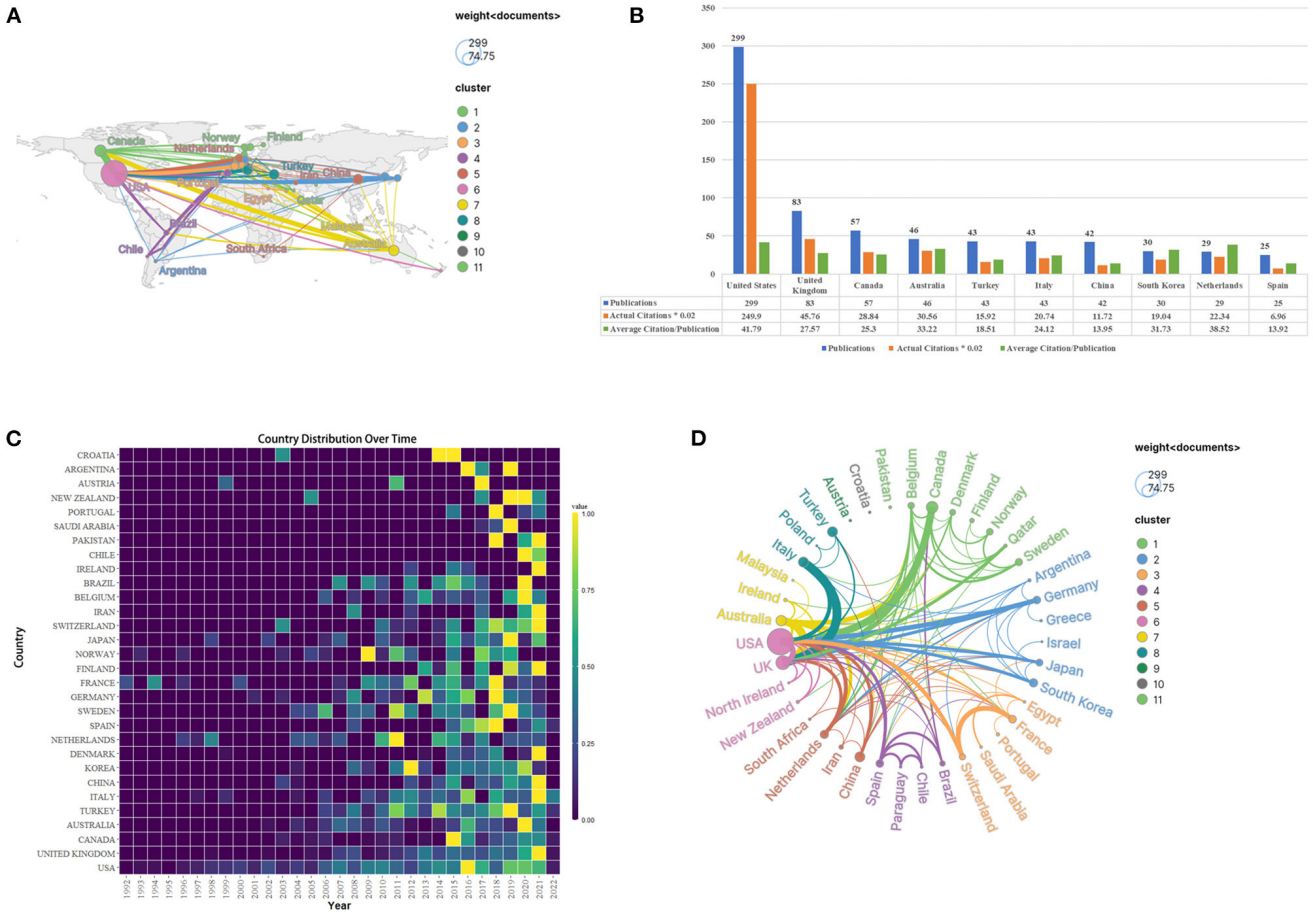
The information on contributive countries of the rehabilitation of rotator cuff injury. **(A)** The geographical visualized map of countries. **(B)** The number of publications, total citations and citations per year in the top 10 countries. **(C)** The heatmap of country distribution over time from 1992 to 2022. **(D)** The cooperative network visualized map of countries.

The geographical map of active research countries generated by Scimago Graphica shows that a total of 48 countries contributed to the field of rehabilitation of RCI (Figure 3A). The top 10 most productive countries are listed in Figure 3B based on the number of publications, with the US producing the most publications (*n* = 299, 37.6%) between 1991 and 2021, followed by the United Kingdom (UK) (*n* = 83, 10.4%), Canada (7.2%), Australia (*n* = 46, 5.8%), Turkey (*n* = 43, 5.4%). Among the top 10 countries with high outputs, only Turkey and China were developing countries while others were developed countries. The number of citations in the US was also the highest (*n* = 12,495), which was roughly 5.5 times that of the second-ranked UK (*n* = 2288). In terms of average citations, the US has the highest average number of citations (*n* = 41.79). A heatmap of national publication trends from 1992 to 2022 was created using the “bibliometrix” and “ggplot” packages in the R Project for Statistical Computing (Figure 3C). The results in Figure 3C suggest that less research was published in this field before 2008, with only a few countries publishing relevant studies. From 2008 to 2021, the top-ranked countries (US, UK, Canada, Australia, Turkey, Italy, and China) became active, and the overall number of publications showed an upward trend. In the collaborative network generated by Scimago Graphica (Figure 3D), the US holds the greatest collaborative network with different countries. The strength of cooperation was stronger between the US and Canada, the UK and Italy, and France and Switzerland, demonstrating there was a strong willingness to cooperate among these regions.

### Contributions of institutions

**Figure 4:**
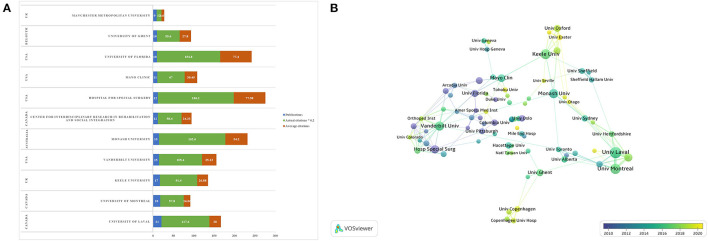
The information on contributive institutions of the rehabilitation of rotator cuff injury. **(A)** The number of publications, total citations and citations per year in the top 10 institutions. **(B)** The co-network visualized map of institutions.

A total of 1,257 institutions have published literature on the rehabilitation of RCI. As shown in Figure 4A, the institutions that published the most papers were the University of Laval (*n* = 21), followed by the University of Montreal (*n* = 18), and Keele University (*n* = 17). Among the top 10 institutions, 4 are from the US and 3 are from Canada, indicating that the research in this field in North America is at the leading level. Additionally, as shown in Table 2, among the top 10 institutions, The Hospital for Special Surgery has the highest total and average citations (931 and 77.58, respectively). The minimum number of institutional publications was set as 4, and 89 institutions were selected for institutional co-authorship analysis *via* VOSviewer software (Figure 4B). Among them, 89 institutions formed the largest institutional co-authorship network, which were divided into 20 clusters. The green cluster is the largest one including 13 institutions, which was centered at Vanderbilt University. The light green cluster ranked second and was concentrated at Mayo Clinic. The top 3 institutions with the largest total link strength (TLS) were the University of Laval (TLS = 34), the Center for Interdisciplinary Research in Rehabilitation and Social Integration (TLS = 31) and the University of Montreal (TLS = 29), indicating that they had participated in more collaborations than others. In addition, the three institutions mentioned above have reported relevant research mainly in recent 5 years, which shows that these institutions were the leaders in this field.

**Table 2:** Top 10 contributive institutions with publications on the rehabilitation of RCI.

**Rank**	**Country**	**Institution**	**Publications**	**Actual citations**	Actual citations *0.2	**Average citations**
1	Canada	University of Laval	21	588	117.6	28.00
2	Canada	University of Montreal	18	289	57.8	16.06
3	UK	Keele University	17	457	91.4	26.88
4	USA	Vanderbilt University	15	527	105.4	35.13
5	Australia	Monash University	15	813	162.6	54.20
6	Canada	Center for Interdisciplinary Research in Rehabilitation and Social Integration	12	292	58.4	24.33
7	USA	Hospital for Special Surgery	12	931	186.2	77.58
8	USA	Mayo Clinic	11	335	67	30.45
9	USA	University of Florida	10	774	154.8	77.40
10	Belguim	University of Ghent	10	278	55.6	27.80

RCI, rotator cuff injury.

### Contributions of journals

**Figure 5:**
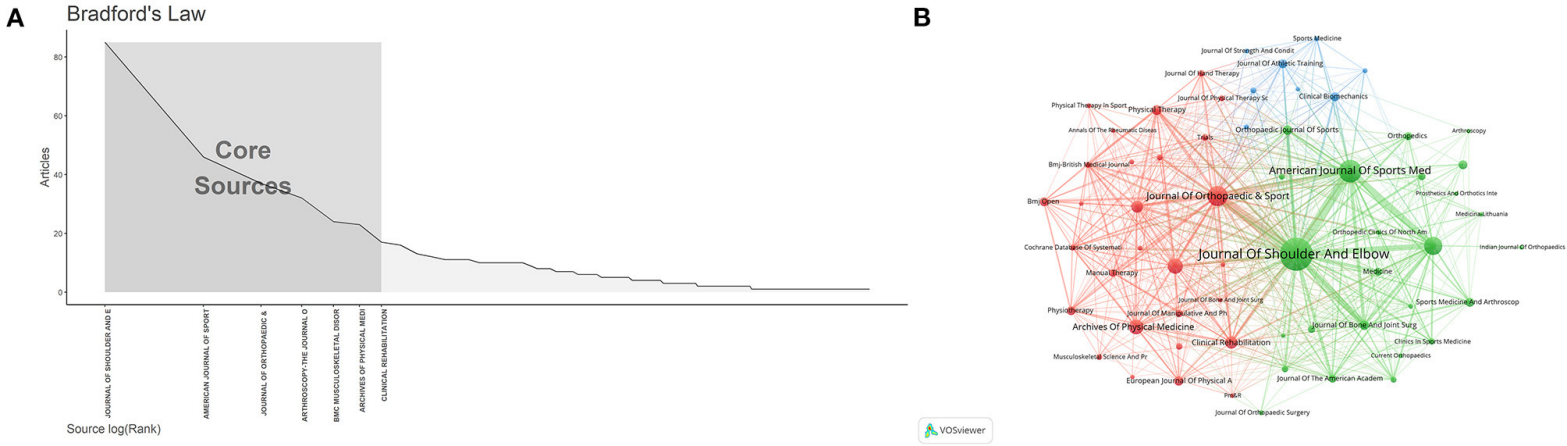
The related information of journals on the rehabilitation of rotator cuff injury. **(A)** The Bradford's Law for journals. **(B)** The citation network of journals.

Between 1991 and 2021, papers related to the rehabilitation of RCI were published in 218 journals. Among the top 10 productive journals listed in Table 3, the Journal of Shoulder and Elbow Surgery had the most publications (*n* = 85), followed by the American Journal of Sports Medicine (*n* = 46) and the Journal of Orthopedic & Sports Physical Therapy (*n* = 37). Bradford's law can be used to identify core journals in different fields, which defines core journals as those that publish more than one-third of all relevant literature (20). Therefore, there are 7 core journals and 211 non-core journals in the current research field (Figure 5A). In terms of citations and average citations, the Journal of Shoulder and Elbow Surgery had the highest citations (*n* = 3,207), followed by the American Journal of Sports Medicine (*n* = 2,439). The British Journal of Sports Medicine had the highest average citations (*n* = 71.21), which also had the highest IF among the top 10 journals (IF = 18.473). According to the WOS journal impact factor quartile assessment, around 25% of the documents were published in Q1 (21) and 70% of the top 10 journals were published in Q1.

**Table 3:** Top 10 journals contributed to publications on the rehabilitation of RCI.

**Rank**	**Journal**	**Publications**	**IF (2021)**	**JCR**	**Citations**	**Average citations/publication**
1	Journal of Shoulder and Elbow Surgery	85	3.507	Q2	3,207	37.73
2	American Journal of Sports Medicine	46	7.01	Q1	2,439	53.02
3	Journal of Orthopedic & Sports Physical Therapy	37	6.276	Q1	1,638	44.27
4	Arthroscopy: the Journal of Arthroscopic and Related Surgery	32	5.973	Q1	1,935	60.47
5	BMC Musculoskeletal Disorders	24	2.562	Q3	494	20.58
6	Archives of Physical Medicine and Rehabilitation	23	4.06	Q1	637	27.70
7	Clinical Rehabilitation	17	2.884	Q2	513	30.18
8	British Journal of Sports Medicine	16	18.473	Q1	1140	71.25
9	Physical Therapy	12	3.679	Q1	757	63.08
10	European Journal of Physical and Rehabilitation Medicine	11	5.313	Q1	113	10.27

RCI, rotator cuff injury; IF, impact factor.

VOSviewer software was used to create a visualized map of the co-cited analysis for journals. The result from Figure 5B shows the journal co-citation network with 218 nodes. The size of the node represents the status of journals and the number of published papers. As shown in Figure 5B, all journals are divided into 3 clusters. The red cluster contains journals such as the Archives of Physical Medicine and Rehabilitation and the Journal of Orthopedic & Sports Physical Therapy, representing journals related to rehabilitation medicine. The green cluster containing journals such as the Journal of Shoulder and Elbow Surgery and the American Journal of Sports Medicine represents journals related to surgery.

### The highly cited publications

The top 10 most cited papers in the field of rehabilitation of RCI are shown in Table 4. These studies were conducted between 1995 and 2013, and each paper was cited at least 218 times. Among the 10 publications, there were 5 original articles, 4 reviews and 1 Protocol. The highest-citation publication (*n* = 406) is “Current Concepts in the Rehabilitation of the Overhead Throwing Athlete” in the American Journal of Sports Medicine in 2002, which illustrated that the rehabilitation of RCI among overhead throwing athletes should attend a structured method concentrating on controlling inflammation, rebuilding muscle balance, enhancing the flexibility of soft tissue (22).

**Table 4:** The top 10 papers with the most citations.

**Rank**	**Title**	**Journal**	**Author (Year)**	**Document type**	**Citations**
1	Current Concepts in the Rehabilitation of the Overhead Throwing Athlete	American Journal of Sport Medicine	Wilk KE (2002)	Review	406
2	Surgical Repair of Chronic Rotator Cuff Tears: A Prospective Long-Term Study	The Journal of Bone & Joint Surgery	Cofield RH (2001)	Article	382
3	Clinical implications of scapular dyskinesis in shoulder injury: the 2013 consensus statement from the ‘scapular summit'	British Journal of Sport Medicine	Ben Kibler W (2013)	Review	334
4	Shoulder Function and 3-Dimensional Scapular Kinematics in People with and without Shoulder Impingement Syndrome	Physical Therapy	Mcclure PW (2006)	Article	289
5	Acupuncture for shoulder pain	Cochrane Database of Systematic Reviews	Green S (2005)	Review	289
6	Platelet rich plasma in arthroscopic rotator cuff repair: a prospective RCT study, 2-year follow-up	Journal of Shoulder and Elbow Surgery	Randelli P (2011)	Article	273
7	Exercise in the treatment of rotator cuff impingement: A systematic review and a synthesized evidence-based rehabilitation protocol	Journal of Shoulder and Elbow Surgery	Kuhn JE (2009)	Protocol	254
8	Debridement of degenerative, irreparable lesions of the rotator cuff	The Journal of Bone & Joint Surgery	Rockwood CA (1995)	Article	250
9	Shoulder Muscle Activity and Function in Common Shoulder Rehabilitation Exercises	Sports Medicine	Escamilla RF (2009)	Review	218
10	Shoulder kinematics with two-plane x-ray evaluation in patients with anterior instability or rotator cuff tearing	Journal of Shoulder and Elbow Surgery	Paletta GA (1997)	Article	218

### Contributions of authors

**Figure 6:**
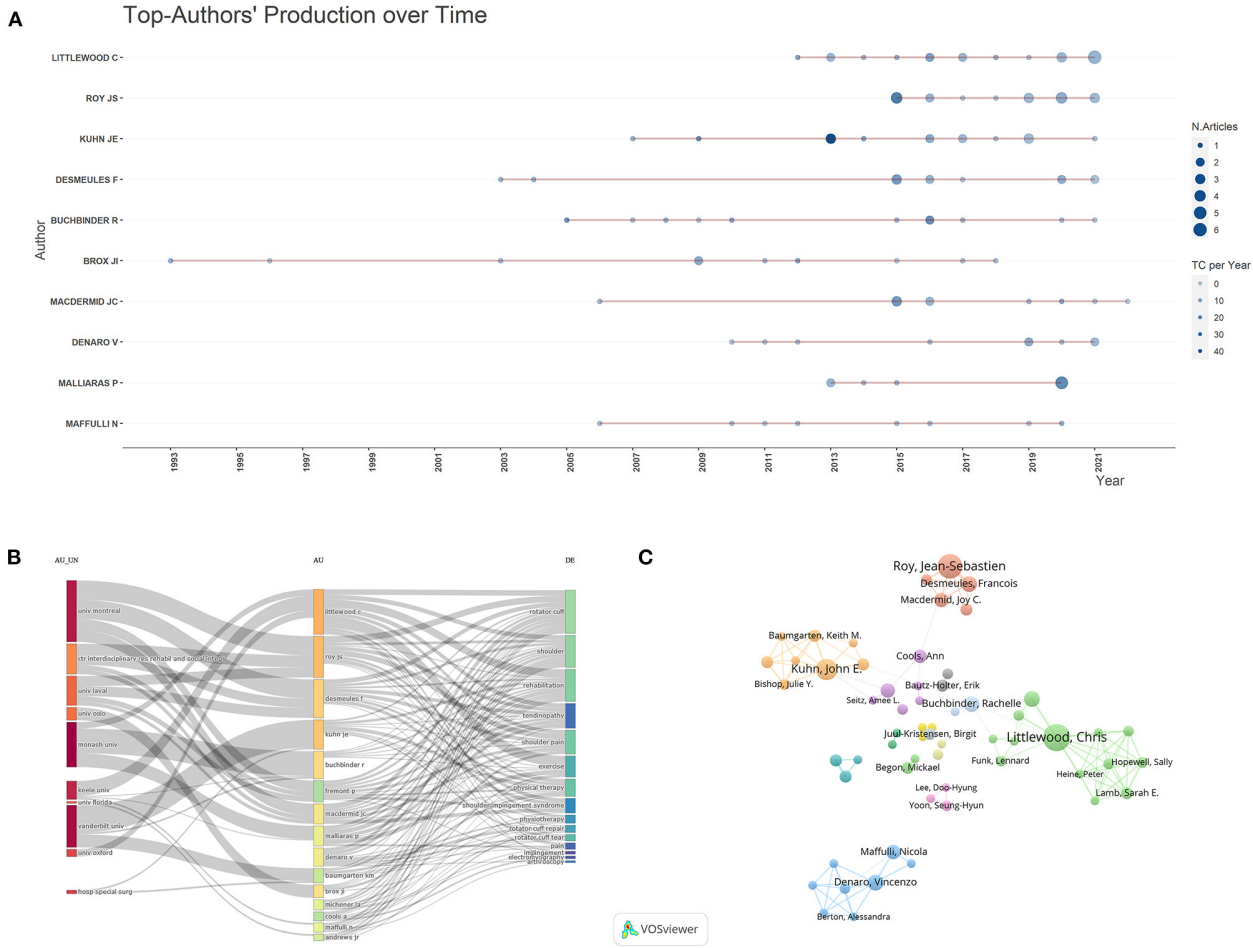
The top 10 authors and cooperative network on rehabilitation of rotator cuff injury. **(A)** The top-10 authors' production over time. **(B)** The three-dimensional plots over the authors, affiliations and research directions. **(C)** The co-network visualized map of authors.

From 1991 to 2021, a total of 3,185 authors published papers on the rehabilitation of RCI. The top 10 prolific authors are presented in Table 5. The author with the largest number of published papers is Littlewood et al. (23) (20 papers) from Keele University, the UK. Kuhn et al. (24) from Western University in Canada has the highest citation and average citation, with 1,007 and 67.13 times, respectively. Both Littlewood et al. (23) and Kuhn et al. (24) had the highest H-index with 10. According to the results in Figure 6A, it can be inferred that the articles published by authors from Canada were mainly after 2013. Roy JS, Desmeules F, and Macdermid JC ranked 2 to 4 in outputs and collaborated many times in the same research group at the University of Montreal (Figure 6B). Additionally, there were two authors from the University of Monash, and the others were from various institutions around the world. We selected 70 items for visual analysis of co-authorship analysis for authors in VOSviewer software. The network of co-authorship for authors was composed of 70 authors, which was divided into 24 clusters (Figure 6C). The largest red cluster contained 13 authors. The green cluster (8 authors), centered on Denaro Vincenzo, is a closely-collaborated research team. Moreover, the third-ranked cluster (blue cluster with 8 authors) is mainly centered in Kuhn et al. (24). The top three authors in total link strength were Littlewood et al. (23) (TLS = 48), Denaro Vincenzo (TLS = 30), and Kuhn et al. (24) (TLS = 28).

**Table 5:** Top 10 contributive authors with publications on the rehabilitation of RCI.

**Rank**	**Author**	**Country**	**Affiliation**	**Publications**	**Citations**	**Average citations/ publication**	**H-index**
1	Littlewood Chris	UK	Keele University	20	449	22.45	10
2	Roy Jean-Sebastien	Canada	University of Laval	18	305	16.94	5
3	Kuhn John E	Canada	Wester University	15	1007	67.13	10
4	Desmeules Francois	Canada	University of Montreal	12	193	16.08	8
5	Buchbinder Rachelle	Australia	Monash University	11	385	35.00	9
6	Brox, Jens Ivar	Norway	University of Oslo	10	232	23.20	9
7	Macdermid Joy C	Canada	Western University	10	206	20.60	8
8	Denaro Vincenzo	Italy	University Campus Bio-medico- Rome	9	142	15.78	7
9	Malliaras Peter	Australia	Monash University	9	209	23.22	8
10	Maffulli Nicola	Italy	University of Salerno	8	177	22.13	7

RCI, rotator cuff injury.

### Bibliometric and visualized analysis of keywords

**Figure 7:**
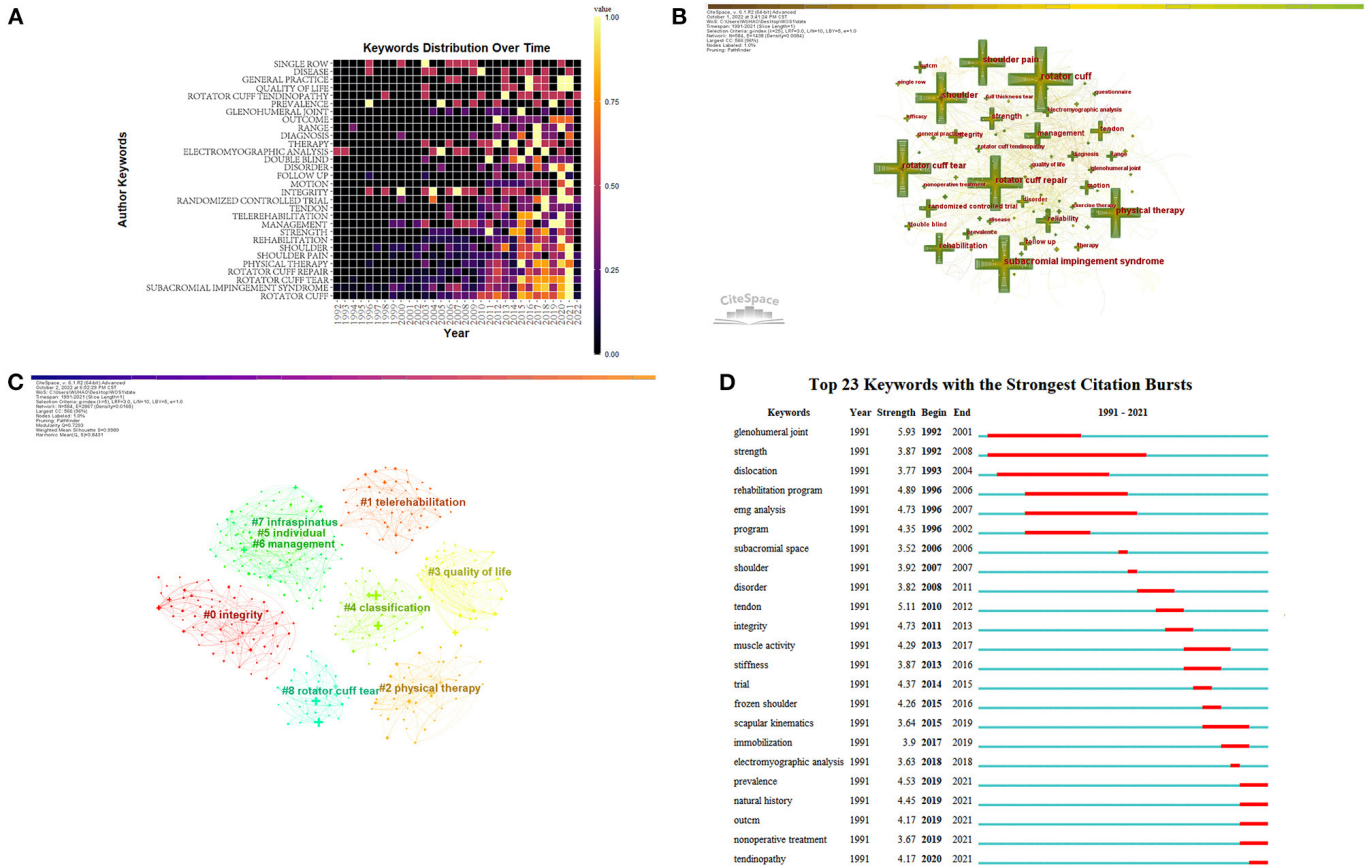
Bibliometric analysis of keywords. **(A)** The heatmap of keywords distribution over time from 1992 to 2022. **(B)** The co-occurrence network map of keywords. **(C)** The co-occurrence cluster map of keywords. **(D)** The top 20 keywords with the strongest citation bursts.

The heatmap of keywords distribution over time was created using the “bibliometrix” and “ggplot” packages in the R Project for Statistical Computing (Figure 7A). Before 1999, research in this field focused on the biomechanics of RCI, and the keywords at this stage were “shoulder pain” and “biomechanics”. Since 2000, a large number of studies have gradually focused on the rehabilitation of RCI, and the keywords at this time were mainly “rehabilitation”, “physical therapy” and “exercise therapy”. After 2010, many studies on the treatment of RCI involving techniques such as injections and ultrasound began to appear, and the keywords at this time mainly increased “injections” and “ultrasonography”.

Citespace was used to analyze the keywords to obtain a co-occurrence network, which contained 584 nodes and 3,718 connections. After merging similar words, a keyword co-occurrence map was obtained. As shown in Figure 7B. The top 20 most frequently used keywords were shown in Table 6, of which the top 5 most frequently used keywords were “rotator cuff” (215 times), “subacromial impingement syndrome” (171 times), “rotator cuff tear” (162 times), “rotator cuff repair” (158 times), and “physical therapy” (158 times). However, not all high-frequency keywords had high centrality at the same time. Therefore, it was necessary to identify research hotspots in combination with keyword centrality. Keywords with high centrality were “electromyography analysis” (centrality = 0.16), “glenohumeral joint” (centrality = 0.15), “strength” (centrality = 0.14), “general practice” (centrality = 0.14), “full thickness tear” (centrality = 0.13), “follow up” (centrality = 0.13). The logarithmic likelihood ratio (LLR) algorithm was used for keyword clusters and a total of 9 clusters (Figure 7C) with a Q value of 0.7293 were generated, of which the largest connected component included 566 nodes, accounting for 96% of the entire network. The mean silhouette value was 0.8847 which was greater than 0.5, indicating that the classification of clusters was reasonable.

**Table 6:** The top 20 most frequently used keywords.

**Number**	**Count**	**Centrality**	**Year**	**Keywords**
1	215	0.02	1996	Rotator cuff
2	171	0.03	1996	Sub-acromial impingement syndrome
3	162	0.11	1995	Rotator cuff tear
4	158	0.1	1995	Rotator cuff repair
5	158	0.03	1998	Physical therapy
6	149	0.02	1996	Shoulder pain
7	121	0.03	1993	Shoulder
8	95	0.06	1997	Rehabilitation
9	71	0.13	1992	Strength
10	71	0.01	2001	Management
11	58	0.09	1998	Reliability
12	44	0.02	1993	Tendon
13	44	0	2009	Randomized controlled trial
14	43	0.07	2003	Integrity
15	38	0.02	1998	Motion
16	35	0.12	1995	Follow up
17	30	0.04	2004	Disorder
18	28	0.11	1997	Double blind
19	27	0.16	2000	Electromyographic analysis
20	26	0.04	1997	Therapy

In Figure 7C, according to the different types of keywords in each cluster, the 9 clusters were divided into 3 categories. The high-frequency keywords that the first kind of cluster included were #2 “physical therapy”, #7 “infraspinatus” and #4 “classification”, which mainly described the importance of conservative treatment for patients with RCI. The second kind of cluster included 0#, 8#, 3#, and 5#, among which the high-frequency keywords were “integrity”, “rotator cuff tear”, “quality of life”, and “individual”, respectively, which represented the rehabilitation of RCI during the perioperative period. The third kind of cluster represented the management model of long-term rehabilitation outside the hospital, including 1# and 6#, among which the keywords were “telerehabilitation” and “management”.

Also, we generated a map of keywords with the strongest citation bursts. The top 23 keywords were listed in Figure 7D, of which “strength” had the longest burst time (1992–2008), which was related to the biomechanical research of the rotator cuff and postoperative rehabilitation. The keywords with the highest burst strength were “glenohumeral joint”, “tendon”, “rehabilitation program”, “integrity”, and “EMG analysis”, which indicated that these words burst frequently in a short period. In addition, the words “prevalence”, “natural history”, “non-operative treatment”, and “tendinopathy” burst in recent years, indicating the frontier of research in this field.

## Discussion

### Main findings of results

The current study aims to summarize the research in the field of rehabilitation on RCI in the past 30 years by using the research method of bibliometrics, and demonstrate its research hotspots and frontiers. A total of 795 studies related to the rehabilitation on RCI were included from the WOS. Littlewood et al. (23) was the most prolific author (20 publications). The US and the University of Laval were the leading country and institution in this field, with 299 and 21 publications, respectively. There was active collaboration between different authors, countries and institutions. Hot topics focused on relatively well-established rehabilitation of RCI such as passive motion or exercise therapy. Hot topics focused on telerehabilitation and artificial intelligence.

This study explains a systematic review of research on the rehabilitation of RCI worldwide over the past 30 years. According to the time of publication, the research in this field can be roughly divided into three stages. As of 2001, rehabilitation of RCI was still in the exploratory stage. Researchers at this stage were primarily exploring treatments to alleviate shoulder pain and limited joint mobility caused by RCI, with rehabilitation as a complementary treatment. For example, one study mentioned that for patients with RCI who do not require surgery, non-steroidal drug therapy is often sufficient and rehabilitation as an alternative treatment may benefit patients (25). Research during this stage focused on the application of rehabilitation to alter the biomechanics of RCI. Many studies supported the positive effect of rehabilitation on ameliorating the instability of the glenohumeral joint in patients with RCI (26, 27). With the continuous development of rehabilitation technology, more and more researchers have begun to pay attention to its importance. According to the results in Table 1 and Figure 2, the number of studies related to the rehabilitation of RCI showed a rapid upward trend after 2015. This may be due to advances in smart technology, which has promoted the application of rehabilitation in RCI. For example, researchers have used computational musculoskeletal modeling to equip patients with RCI with a collaborative robot that could give patients robot-mediated therapy. The robot was able to sense the internal state of patients and reduce the potential risk of the rotator cuff muscle being retorn while treating (28). Additionally, rehabilitation has been used as a conservative treatment for patients with RCI who did not undergo surgery in the past. In recent years, research has focused more on postoperative rehabilitation of RCI. One study has shown that early and delayed postoperative mobilization has positive effects on pain relief and increased joint mobility after rotator cuff repair. This may be due to advances in surgical techniques that allow more patients to undergo arthroscopic surgery, thereby increasing the need for postoperative rehabilitation (29). With the development of 5G technology, it is expected that research related to intelligent rehabilitation may further explode, and the application of intelligent wearable devices associated with RCI may increase and become a reality. Therefore, we suggest that researchers focus not only on medical research but also on interdisciplinary cooperation, especially the study of the integration of medicine and engineering, which may promote the further development of rehabilitation of RCI in the future.

The results of Figures 3, 4 show that countries in economically developed regions such as North America, Europe, and Australia, as well as research institutions located in these countries, are dominant in the field of rehabilitation of RCI, which indicates that the development of scientific research is closely related to economic level, research group and atmosphere. Nevertheless, developing countries in Asia represented by China and Turkey have also become active in this field since 2010. However, it should be noted that except for the US, which has a relatively wide network of scientific research cooperation in the world, other research cooperation is mostly realized between different institutions in the same country. As a result, there is still a relative lack of collaboration between countries or institutions on a global scale. In the future, it is necessary and important to research technology-based rehabilitation on a global scale, because multi-center, large-sample and high-quality studies covering different ethnic groups can provide more reliable evidence for clinical practice.

As for the visual analysis of journals, according to the results in Table 3, the top 10 high-production journals include 303 papers in total, accounting for about 38% of the total number of publications in this field, which indicates that the papers related to the rehabilitation of RCI were widely published in different journals, and the cluster of core journals has not yet been presented. However, it is worth noting that the JCR partitions of the top 10 active journals all belong to Q1 and Q2, which means that these journals are of relatively high quality. Authors with research interests in this field can focus on these journals and consider them when submitting their work for publication. As shown in Figure 5B, the cooperation network of journals related to this field presents two relatively stable clusters, which respectively represent the journals associated with physical and rehabilitation medicine and surgery. As the research in this field develops toward intelligent technology, perhaps more publications will be published in medical and science-related journals in the future and researchers can also consider and pay attention to these journals.

In terms of the visualized results for authors in this field, it is obvious that the top 10 prolific authors had almost no output before 2003. However, we looked at papers published before 2003 and found that research in this area focused on the efficacy of manual therapy for RCI. For example, the research team at Lastayo PC noted that manual passive range-of-motion exercises were beneficial to patients with RCI (30). Between 2003 and 2010, researchers in the field focused primarily on the impacts of exercise-based rehabilitation on RCI. For example, the group by Reed D concentrated on the effects of exercise therapy on shoulder muscle activation in patients with RCI (31). Additionally, Mcclure et al. investigated the effects of exercise therapy on three-dimensional kinematics of the shoulder after RCI (32, 33). After 2010, researchers in this field paid more attention to injection therapy and physical therapy. For example, the research teams of Prodromos CC, Hamid MSA, and Sazlina SG and Xiang focused on platelet-rich plasma (PRP) for the treatment of rotator cuff tendinopathy (34–36). In addition, the collaboration between the research teams of Roy JS, Desmeules F, and Macdermin JC occurred mainly after 2013, and they studied the efficacy of ultrasound and transcutaneous electrical nerve stimulation in the treatment of RCI (37, 38). Similarly, Belley et al. investigated the effects of transcranial direct current stimulation in patients with rotator cuff tendinopathy (39). In recent years, many studies have also focused on the impact of wearable devices, telerehabilitation, and machine learning on the monitoring and treatment of patients with RCI (40–42), which to a certain extent can represent current research hotspots.

### Keywords analysis

The extracted keywords were divided into 9 clusters. According to the different types of keywords in each cluster, the 9 clusters were divided into 3 categories, the first of which was about the conservative treatment of RCI, which is usually applied to patients with chronic injuries, where the tendon has not retracted significantly, and the rotator cuff structure is largely intact. This type of cluster includes 2# “physical therapy”, 7# “infraspinatus”, and 4# “classification”. In addition, this type of cluster mainly focuses on non-surgical treatment of RCI, including physical therapy, exercise therapy, and injection therapy. For example, Kuhn et al. (24) performed physical therapy on patients with non-traumatic full-thickness rotator cuff tears. After 2 years of follow-up, 75% of the patients had reduced shoulder pain and regained function (24). Also, exercise therapy and injection therapy are often used in conservative treatment. After years of development of injection therapy, a variety of compound injections have been developed, such as amino acid collagenase compound and autologous adipose-derived regenerative cells, which can promote the repair of the rotator cuff (24, 43–46). Moreover, many physical therapies such as dry needling, ultrasound, and laser therapy have still been shown to be feasible and effective in patients with RCI (47–50). Also, technological progress has promoted the automation of medical technology, which is not only manifested in surgery, but also in the field of physical therapy assisted by robots, which has proven to be a promising development model (28).

The second type of cluster was related to perioperative rehabilitation of RCI, which included 0# “integrity”, 8# “rotator cuff tear”, 3# “quality of life”, and 5# “individual”. Surgery is usually reserved for acute injuries to the rotator cuff with compromised structural integrity. For the surgical repair of the rotator cuff, it has undergone a development process from open surgery to minimally invasive surgery (51). Therefore, it is extremely crucial to choose an appropriate rehabilitation plan for different surgical methods. Ghodadra et al. proposed corresponding rehabilitation strategies for open, mini-open, and arthroscopic surgery to maximize the effect of rehabilitation (52).

The rapid development of arthroscopic techniques has enabled the firm repair of the rotator cuff also with minimally invasive techniques. A large number of studies related to ERAS appear. For example, Duzgun et al. compared the effectiveness of accelerated rehabilitation (starting 4 weeks before surgery and 3 weeks after surgery) and conventional rehabilitation protocol (starting at 6 weeks post-operatively), and the results found that the accelerated rehabilitation program was better than the conventional program in pain control and the improvement of limb function (53).

The third cluster was the management model of long-term out-of-hospital rehabilitation, which included 1# “telerehabilitation” and 6# “management”. Rehabilitation of RCI is a long-term process. To ensure the quality of rehabilitation and compliance of patients, medical staff have proposed a variety of management methods. For example, many research institutions have developed a telerehabilitation system to provide remote guidance for patients undergoing rotator cuff arthroscopic surgery. In a recent study, telerehabilitation was used in patients with sub-acromial impingement syndrome after arthroscopic surgery. Patients in the intervention group received an individualized rehabilitation program through a web application. The application allowed the physical therapists to generate videos, images and parameters of the exercise plan and email them to the patients. Physical therapists supervised and trained patients through online videos. The results showed that telerehabilitation was no less effective than traditional face-to-face rehabilitation (54). Similarly, another study used a telemedicine platform (JeffConnect) for regular rehabilitation of patients undergoing rotator cuff repair through arthroscopy. Compared with the patients who were regularly rehabilitated offline under face-to-face guidance, it was found that the treatment and follow-up effects achieved by the two rehabilitation interventions were similar. However, telerehabilitation takes less time and is more convenient for both the patients and the physical therapists (55). Alternatively, a long-term self-management training program developed by physical therapists for rotator cuff tendinopathy could also achieve good results, but it requires good patient compliance (23). In addition, based on the advancement of science and technology in the information age, the application of virtual vision systems represented by virtual reality to telerehabilitation seems to be a new solution, and the related research is increasing (56). Therefore, how to prolong the patient's out-of-hospital rehabilitation is still a question worthy of discussion.

Also, the results of Figure 7D suggest that “glenohumeral joint”, “tendon”, “rehabilitation program”, “integrity”, and “EMG analysis” have high burst intensity, suggesting that it is a research hotspot in this field. At present, the research on rotator cuff injury has not only been limited to the local muscle-tendon tissue but also focused on the important structures around the rotator cuff. Different from the previous assessment methods based on images, the assessment of rotator cuff injuries now combines other techniques such as muscle electrophysiology and provides objective electrophysiological data to guide the formulation of more accurate and personalized rehabilitation training programs. For example, Reinold et al. investigated EMG changes in the rotator cuff during different shoulder movements to determine which movement pattern was most effective for enhancing strength and restoring the function of the affected limb, to formulate the most effective rehabilitation program (57).

### Strengths and limitations

To our knowledge, this is the first study to use the co-occurrence and co-citation analysis methods by Citespace, VOSviewer, Scimago Graphica software, and R Project to perform bibliometric analysis and visual display of the rehabilitation of RCI from hot spots, co-cited references, and cooperation among authors, countries, and institutions.

Although this study is the first bibliometric study based on the rehabilitation of RCI, there are some limitations. First, although WOS is an authoritative database containing various publications that fit the subject of this study, one database cannot identify all studies. Therefore, the search results of this study may be different from the actual number of publications. Second, only English literature was retrieved and included in this study, and therefore important studies in other languages may be ignored. Third, this study only retrieved studies published as of December 2021, and articles published in 2022 were not included in this study, which may also affect the accuracy of this study. Therefore, there are some suggestions for future research can be made. In future research, more databases such as PubMed and Scopus can be included when extracting publications. Moreover, publications in multiple languages such as Chinese can also be included. Furthermore, future studies could also expand the range of publication time, and these suggestions could increase the reliability of the studies.

## Conclusion

Based on bibliometric analysis, the current study shows a steady upward trend in the total number of publications since the beginning of the 20^th^ century. Countries and institutions in North America, Europe, Australia, and other developed regions have played a leading role in the field of rehabilitation of RCI, but there is still a relative lack of co-operation among countries on a global scale. Most of the related research in this field was published in internationally influential journals. In addition to the relatively well-established rehabilitation on RCI such as passive motion or exercise therapy, with the development of science and technology, the current research focus tends to develop toward telerehabilitation and artificial intelligence. These findings may help future researchers better understand the hotpots in the field of rehabilitation of RCI.

## Data availability statement

The raw data supporting the conclusions of this article will be made available by the authors, without undue reservation.

## Author contributions

YH and LW contributed to the design of the study and prepared the manuscript. YH, LHe, and LHu conducted the literature search. YW, XL, and XZ conducted the software to generate the figures. All authors contributed to the manuscript revision, read, and approved the submitted version.
